# The Abuse of Mineral Waters

**Published:** 1887-08

**Authors:** Germain See

**Affiliations:** Hotel Dieu, Paris


### ABSTRACT OF A CLINICAL LECTURE ON THE ABUSE OF MINERAL WATERS.

BY PROFESSOR GERMAIN SEE, AT HOTEL DIEU, PARIS.

[From the *Journal de Médecine et de Chirurgie Practiques*. Translated by Professor Frank Billings].

WATERS naturally gaseous, acidulated or charged with carbonic acid, contain the latter in a proportion from 50 to 200 and 300 volumes of gas in a litre of water; when the salts contained in the waters are inferior to 1 per 1000, whether iron, calcium or bicarbonate of sodium, they merit only the name gaseous or acidulated, and have no virtue but that of carbonic acid gas.

This gas induces in the stomach an excitement of the mucous membrane and gastric nerves, producing an immediate sense of exhilaration and a certain stimulation of appetite, which oftentimes proves detrimental to the patient. Taken in large amounts and used continually, all of these waters introduce a new quantity of gas into the stomach, which mixes with that which already exists in the form of atmospheric air in the stomach, and gas of decomposition, especially carbonic acid, in the intestine; this gas materially aids in causing a sluggishness of the stomach, an atony with or without painful spasms. When these waters have a sodium base, they present the great advantage of stimulating the secretory function; but of what avail is a water feebly mineral and strongly carbonic, especially an artificial carbonic water, unless to augment the tympanism? The moving action of the gas upon the stomach or intestine cannot be estimated; for, taken in large quantities, it hinders rather than facilitates the peristalsis. The most incontestable effect of gaseous mineral waters is their action in augmenting the urinary secretion. The reason for this diuretic action is that under the influence of active gas it establishes a gastric mucous hyperæmia which accelerates the resorption of the mineral water. This is without doubt why alcoholic and gaseous drinks, like champagne, are more intoxicating than those which are not gaseous. These diuretics are generally pernicious, for they excite the renal secretion more than the vesical excretion; now renal irritation has no advantage; waters have power, for example in gravel, by carrying away the sand from the tubes of the kidneys, but, at the same time, excessive secretion is to be feared, because it may produce artificial lesions. If the kidneys are injured in any way, the use of water is dangerous. In all cases of nephritis, interstitial or parenchymatous; in the albuminuries, from all causes, mineral waters are not alone contra-indicated, but are disastrous. Professor Sée gave two examples in which the use of diuretics in an albuminous, gouty nephritis, resulted in death. It is necessary then to avoid the ill use of gaseous waters, which are generally more agreeable than efficacious.

Among mineral waters presenting the most incontestable advantages, the alkaline sodium waters must be placed, *and appear to be effective in most cases of dyspepsia.* Of these, Vichy waters rank first, chiefly by the simplicity of their composition; they dissolve at once in the stomach by the intervention of the gastric acids. But clinical experience demonstrates that if the amout of mineral water taken is very considerable, if taken during or after a meal, especially if it acts upon a weak or dilated stomach, far from facilitating digestion, a veritable putrid fermentation is produced; as when one tries to digest an albuminous body with a neutral gastric substance.

Alongside of numerous indications, there are contra-indications, chiefly in flatulent gastralgia, in simple intestinal debilities or in hæmorrhoides; they are plainly injurious in muco-membranous enteritis as well as in intense and serious dilatations of the stomach.

It is then principally in dyspepsias, called chronic, that Vichy water is indicated; but in all cases it should be taken half an hour or an hour before eating; thus preparing and provoking the gastric secretion; taken at meal time it loses all of its advantages.

For habitual drinking, Professor Sée considers tea the most digestive form; a light infusion, taking at least one-half litre and at a high temperature; for the noonday meal its advantages over wine are numerous; it does not ferment; it contains only traces of tannin, whilst coffee contains infinitely more, which coagulates the albumen. The aërated drinks, by the exciting impression they produce upon the stomachic mucous membrane, makes the aliments of the stomach rapidly disappear, and submits them also to the action of the intestino-pancreatic juice. Some patients can digest only by the aid of hot drinks.

An important remark relating to alimentary drinks is that distilled drinks, diluted in water, succeed often much better than wine. There are patients who cannot bear wine without complaining of acidity, or of sensations of hot iron, and who can take rum or brandy with water added, especially after combustion of these liquors.

As a light purgative for dyspeptics, Professor Sée habitually prescribes a preparation composed of equal parts of sublimed sulphur, cream of tartar and calcined magnesia; one spoonful in coffee once or twice a day; *i.e.*, two or three grammes of each of these laxative powders.

